# Evaluation of electrosynthesized reduced graphene oxide–Ni/Fe/Co-based (oxy)hydroxide catalysts towards the oxygen evolution reaction

**DOI:** 10.3762/bjnano.14.34

**Published:** 2023-03-29

**Authors:** Karolina Cysewska, Marcin Łapiński, Marcin Zając, Jakub Karczewski, Piotr Jasiński, Sebastian Molin

**Affiliations:** 1 Laboratory of Functional Materials, Faculty of Electronics, Telecommunications and Informatics, and Advance Materials Centre, Gdańsk University of Technology, ul. Narutowicza 11/12, 80-233 Gdańsk, Poland; 2 Advanced Materials Center, Institute of Nanotechnology and Materials Engineering, Faculty of Applied Physics and Mathematics, Gdańsk University of Technology, ul. Narutowicza 11/12, 80–233 Gdańsk, Poland; 3 National Synchrotron Radiation Centre SOLARIS, Jagiellonian University, ul. Czerwone Maki 98, 30-392 Cracow, Poland

**Keywords:** electrocatalysts, electrodeposition, energy, hydrogen, oxygen evolution reaction

## Abstract

In this work, the specific role of the addition of graphene oxide (GO) to state-of-the-art nickel–iron (NiFe) and cobalt–nickel–iron (CoNiFe) mixed oxides/hydroxides towards the oxygen evolution reaction (OER) is investigated. Morphology, structure, and OER catalytic activity of the catalysts with and without GO were studied. The catalysts were fabricated via a two-step electrodeposition. The first step included the deposition of GO flakes, which, in the second step, were reduced during the simultaneous deposition of NiFe or CoNiFe. As a result, NiFe-GO and CoNiFe-GO were fabricated without any additives directly on the nickel foam substrate. A significant improvement of the OER activity was observed after combining NiFe with GO (OER overpotential η(10 mA·cm^−2^): 210 mV) compared to NiFe (η: 235 mV) and GO (η: 320 mV) alone. A different OER activity was observed for CoNiFe-GO. Here, the overall catalytic activity (η: 230 mV) increased compared to GO alone. However, it was reduced in comparison to CoNiFe (η: 224 mV). The latter was associated with the change in the morphology and structure of the catalysts. Further OER studies showed that each of the catalysts specifically influenced the process. The improvement in the OER by NiFe-GO results mainly from the structure of NiFe and the electroactive surface area of GO.

## Introduction

Nowadays, the industrial production of hydrogen energy is focused mainly on hydrocarbon reforming, which is a low-efficiency and environmentally unfriendly process [1–2]. As an alternative, water electrolysis using renewable energy sources has recently been extensively studied [3]. The main limitation to the efficiency of this process is primarily the oxygen evolution reaction (OER) due to its sluggish kinetics resulting in a high overpotential and low efficiency [4]. To overcome this problem, robust anode electrode catalyst materials are required. Since the Ru- and Pt-based catalysts used so far for OER are made using limited and expensive metals [5], studies on other catalyst materials are being conducted.

Recently, transition-metal-based materials including nickel, iron, and/or cobalt have become promising catalysts for OER [6–10]. The materials are characterized by relatively low cost and environmentally friendly nature [11]. Even though transition-metal-based catalysts still suffer from low surface areas [12], dissolution and aggregation of metallic phase and metal oxides during the active OER process occurs [13]. Hence, Ni-, Fe- and/or Co-based catalysts have been synthesized as hybrid catalysts with different kinds of conductive carbon materials [14–18]. Recently, graphene (Gr)/graphene oxide (GO) has attracted the attention of many researchers due to its high surface area, significant chemical stability, high electrical conductivity, and high mechanical strength [12,19]. Combining a graphene material with Ni-, Fe- and/or Co-based oxides/hydroxides with high chemical reactivity provides both an effective electron pathway through the catalyst [20] and high specific surface area [21], which is desirable for the OER process [13]. The overall electrocatalytic performance of the hybrid electrode can also be improved by choosing a conductive and/or high surface area substrate, such as porous nickel foam [22–23].

In the literature, some research has been performed to evaluate the OER electrocatalytic performance of hybrid materials of Ni-, Fe- and/or Co-based oxides/(oxy)hydroxides and Gr and/or GO. For example, Wu et al. [13] chemically fabricated metal alloys and their oxides (NiCo, CoFe) with nitrogen-doped graphene (N-rGO/NiCo-NiO-CoO, N-rGO/CoFe-Co_2_FeO_4_) on a glassy carbon electrode (GCE). The N-rGO/NiCo-NiO-CoO and N-rGO/CoFe-Co_2_FeO_4_ catalysts revealed an OER overpotential (η) of 260 mV (Tafel slope: 72 mV·dec^−1^) and 320 mV (65 mV∙dec^−1^) determined at 10 mA·cm^−2^ in 1 M potassium hydroxide (KOH), respectively. In another work, nickel/nickel oxide (Ni-NiO) and cobalt/cobalt oxide (Co-CoO) were chemically synthesized with three-dimensional hierarchical porous graphene (3DHPG) on a GCE [24]. Ni-NiO @3DHPG exhibited an OER onset potential *E*_onset_ of 1.53 V vs RHE, η of 164 mV, and a Tafel slope of 55 mV∙dec^−1^, while Co-CoO@3DHPG revealed an *E*_onset_ of 1.59 V vs RHE, η of 168 mV, and a Tafel slope of 65 mV∙dec^−1^ determined in 1 M KOH. In the work of Xia et al. [20], an efficient OER catalyst of Gr/NiFe layered double hydroxide (LDH) was chemically fabricated on a GCE. The catalyst revealed an OER *E*_onset_ of 1.48 V vs RHE and η of 250 mV determined in 0.1 M KOH. Improved electron transport was provided by the graphene material in the catalyst structure. Enhanced OER catalytic performance was also obtained for electrodeposited NiFe LDH combined with GO on nickel foam (GO-NiFe-LDH) [12] and NiFe LDH combined with reduced graphene oxide (rGO) on nickel foam (NiFe-LDH/RGO) [21]. The OER η was determined to be 119 mV and 150 mV determined at 10 mA·cm^−2^ in 1 M KOH for GO-NiFe-LDH and NiFe-LDH/RGO/NF, respectively. The efficient OER was associated with the presence of the electron interaction between the metal and graphene.

The literature presents the possibility of improving OER performance of the electrode by combining Fe-, Ni- and/or Co-based oxides/hydroxides and GO instead of the Ru- and Pt-based catalysts used so far for OER. There is a lack of literature reports presenting some discussions and determining the specific role of the addition of graphene to the state-of-the-art NiFe and/or CoNiFe-based oxide/hydroxides. Moreover, most of the performed studies were focused mainly on chemically synthesized catalysts, which usually required post-processing and some additives (e.g. Nafion) to form an ink to produce an OER electrode. This, in turn, significantly affects the final structure and electrocatalytic performance of the electrode.

Therefore, in this work, the influence of the addition of GO to NiFe and CoNiFe oxides/(oxy)hydroxides catalysts towards the OER was studied. NiFe, CoNiFe, NiFe-GO, and CoNiFe-GO were synthesized by electrodeposition directly on nickel foam. The process made it possible to fabricate OER electrodes with reduced GO and without any additives that could interfere with the structural and electrochemical measurements. The effects of the addition of GO to NiFe and CoNiFe on their morphological, structural, and OER electrocatalytic properties were studied. The role of GO and metallic species in the OER electrocatalytic process is discussed. The fabricated GO-NiFe reveals excellent catalytic performance towards the OER, that is, higher than a state-of-the-art NiFe catalyst measured in alkaline environment.

## Results

### Electrosynthesis and morphology of the deposits

**Figure 1:**
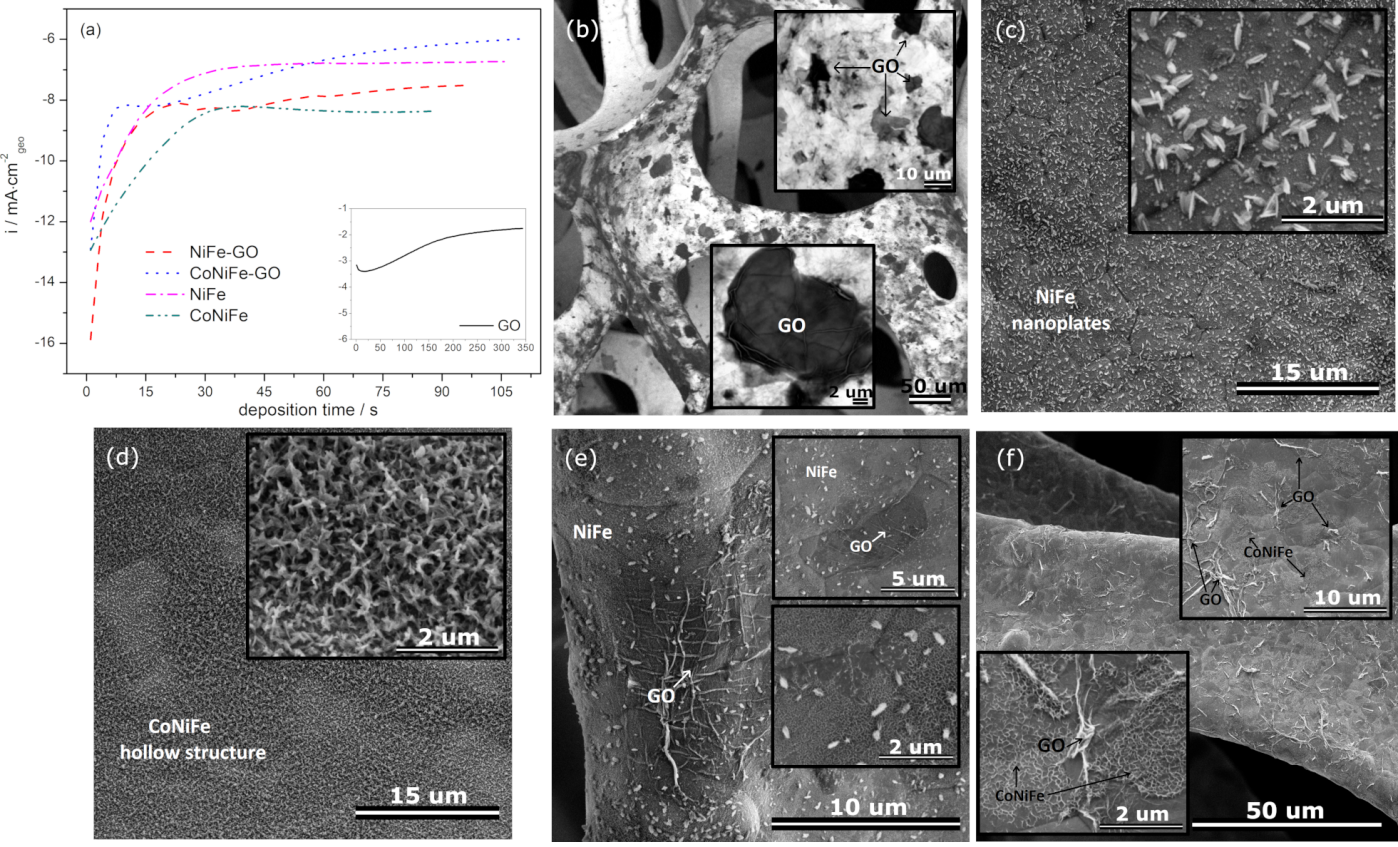
Chronoamperometric graphs recorded during electrochemical deposition of the catalysts on nickel foam (a), SEM images of GO (b), NiFe (c), CoNiFe (d), NiFe-GO (e), and CoNiFe-GO (f) deposited on nickel foam.

The catalysts under investigation were synthesized by electrodeposition onto the surface of nickel foam. The chronoamperometric graph recorded during the deposition is presented in Figure 1a.

Each synthesis (except that of GO) began with a fast increase of the cathodic current, which is associated with the formation of the new catalyst phase on the surface of the substrate [25]. Afterwards, the current density tended to stabilize for NiFe and CoNiFe, which may be associated with the steady-state formation of the catalyst film on the metallic surface. The addition of cobalt to NiFe resulted in a lower overall current density during the synthesis process. In the case of the deposition of NiFe and CoNiFe on GO/nickel foam, the specific current density peak appeared after around 8 s and 20 s of the deposition for CoNiFe-GO and NiFe-GO, respectively. Because the metallic films were deposited on the surface of nickel foam already modified with GO, the peak may be associated with the reduction process of the already deposited GO. Afterwards, the current density increased due to the film formation, and then it gradually stabilized over time. A different chronoamperometric trend can be observed in the case of the electrodeposition of GO on the surface of nickel foam (Figure 1a inset). In this case, the cathodic current density decreased during the first 6 s of the synthesis, then it increased and tended to stabilize. The initial drop of the current density may be related to the preparation (e.g., passivation) of the metallic surface for GO deposition. The latter is a typical process in the electrodeposition of conductive films on active metals [26].

The morphology of the deposits was analyzed by scanning electron microscopy (SEM) and is presented in Figure 1b–f. Typical GO flakes regularly distributed over the surface of the nickel foam were successfully obtained after the one-step electrodeposition process (Figure 1b). The structure of the NiFe deposited directly on the substrate was characterized by the nanoflake-like morphology that is common for electrodeposited NiFe (oxy)hydroxides/oxides LDH [27]. The structure of the NiFe after the addition of cobalt (CoNiFe) was characterized by interconnected nanoflakes, which formed a porous 3D structure uniformly distributed over the entire surface of the nickel foam (Figure 1d). The morphology of the catalysts changed after the combination of GO with NiFe and CoNiFe (Figure 1e,f). In each case, the SEM images clearly show the complete coverage of the surface of the GO/Ni foam with the NiFe or CoNiFe. Less nanoplate-like structures of NiFe could be observed around the GO flakes (Figure 1e). The already deposited GO probably inhibited the formation of Ni and Fe species on its surface. Nevertheless, the morphology of NiFe and GO (Figure 1e) is similar to that observed for each of the singly deposited materials (Figure 1b for GO, Figure 1c for NiFe). Different morphologies can be observed in the case of CoNiFe (Figure 1d) and CoNiFe-GO (Figure 1f). Here, the addition of the GO layer induced much more differences in the morphology of the deposits. Deposition of CoNiFe on the GO/Ni foam changed the shape of the GO flakes, with some visible agglomerations (Figure 1f). In contrast, the presence of GO resulted in the formation of a CoNiFe layer, which only remained an interconnected 3D porous material in some areas. Additional SEM images with different magnifications of the morphology of NiFe and CoNiFe after GO addition are presented in Figure S1 and S2, respectively, in Supporting Information File 1.

**Figure 2:**
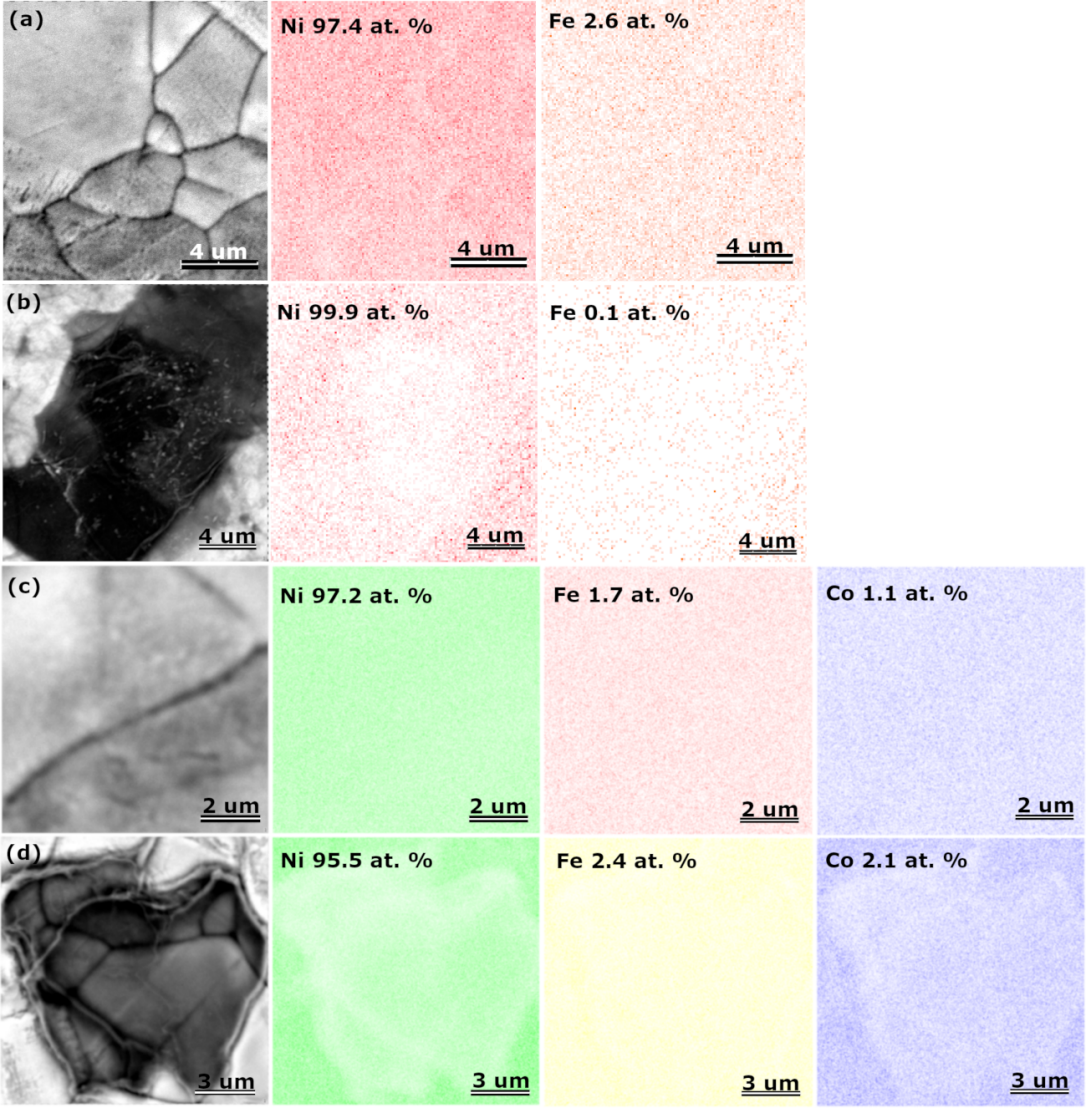
SEM images and corresponding EDX maps of NiFe (a), NiFe-GO (b), CoNiFe (c), and CoNiFe-GO (d) deposited on nickel foam (error ≤ 0.5 atom %).

Figure 2 presents the energy-dispersive X-ray (EDX) maps with corresponding SEM images of the catalysts. The analysis confirms the presence of the following elements in the catalyst structure: Ni and Fe for NiFe and NiFe-GO, and Ni, Fe, and Co for CoNiFe and CoNiFe-GO. A high amount of detected nickel is due to the presence of nickel in the catalyst but also in the nickel substrate. Since an extremely low atom % fraction of iron in NiFe-GO was detected, additional EDX graphs confirming the presence of this element in the catalyst structure has been provided in Supporting Information File 1 (Figure S3). The EDX maps show that then deposition of nickel, iron, and cobalt species is preferable on the surface around the graphene oxide flake. The deposited GO probably inhibited the electrodeposition process of NiFe and CoNiFe on its surface. This may be the reason for the slower stabilization of the synthesis current density observed in the chronoamperograms (Figure 1a).

### X-ray diffraction, X-ray photoemission spectroscopy and X-ray absorption spectroscopy

**Figure 3:**
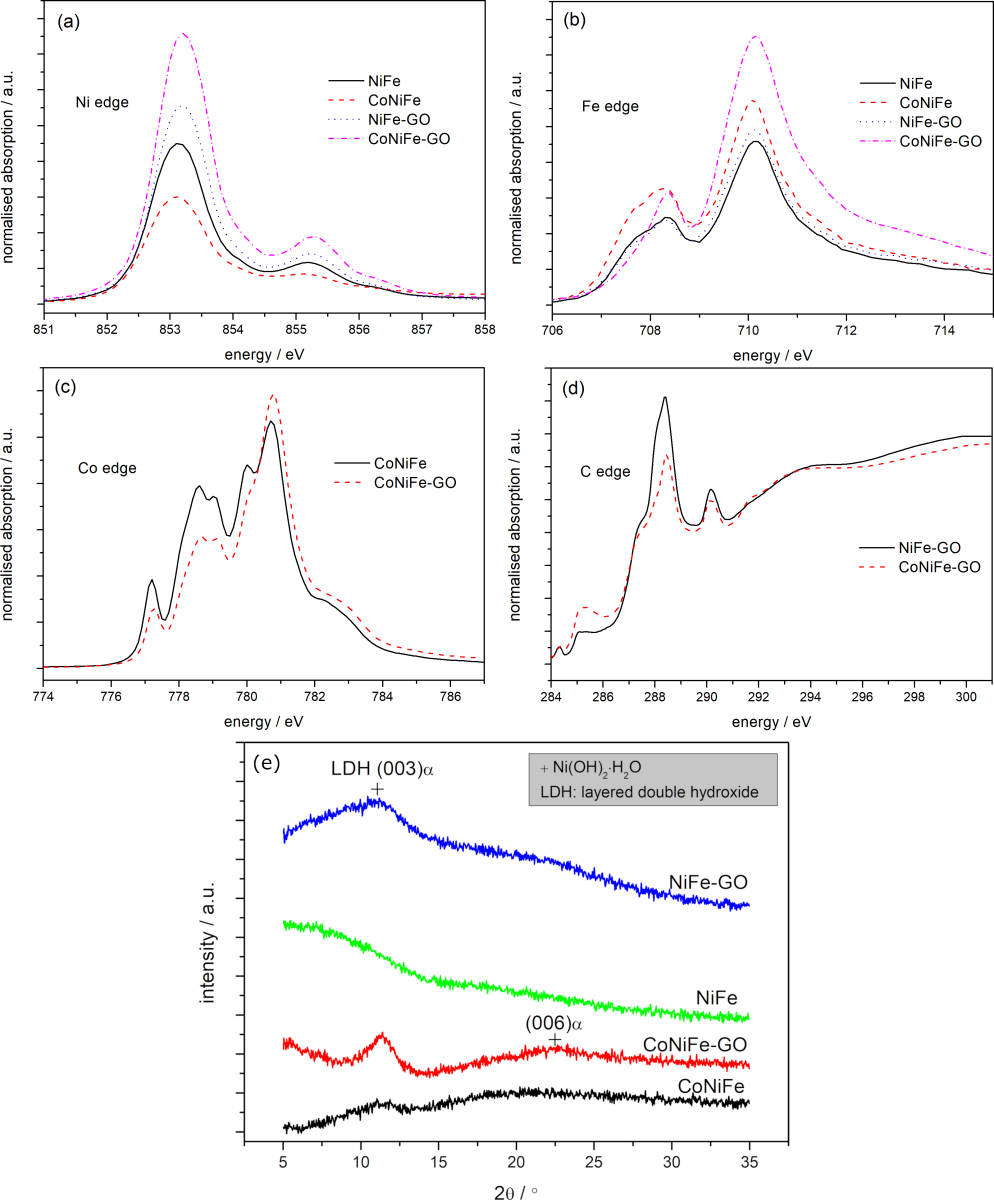
Normalized XAS spectra (a–d) and XRD patterns (e) of NiFe, CoNiFe, NiFe-GO, and CoNiFe-GO.

Figure 3a–d shows the X-ray absorption spectra (XAS) of the L_3_ edge of nickel (a), iron (b), cobalt (c), and carbon (d) in the studied catalysts.

The appearance of a shoulder peak at the L_3_ edge of the nickel (Figure 3a) at 855 eV indicates the presence of oxides in the structure of the catalysts (Ni in a strong crystal field) [28–29]. The shape of the XAS spectra (Ni edge) indicates a similar type of oxides in the structure of the catalysts. The addition of GO to NiFe and CoNiFe intensified both the nickel and iron L_3_ edge peaks, indicating partial electron transfer from nickel and iron to the substitutional GO (carbon) [30]. In the case of the edge of iron (Figure 3b), the XAS spectra indicate the presence of iron atoms in the oxidation state Fe^3+^ in each of the studied catalysts [28–29]. The iron edge peak observed at 707 eV disappeared after the addition of GO to CoNiFe, indicating a change in the structure of the catalyst. However, the type of oxides/hydroxides present in the catalyst structure cannot be determined from the spectra.

A shift of the XAS spectrum and a change in its intensity were observed for the L_3_ edge of cobalt after addition of GO into CoNiFe (Figure 3c). The observed changes indicate charge transfer from cobalt to carbon and the formation of Co–O–C bonds in the catalyst [31]. Moreover, the spectra show that the dominant cobalt species in the studied catalysts were Co^3+^ and Co^2+^ [25].

The L_3_ edge of carbon in NiFe-GO and CoNiFe-GO is presented in Figure 3d. In general, the absorption edges at 285.2 and 293.7 eV correspond to the excitation of electrons in the sp^2^ network into the π^*^ band (C=C) and σ* band (C–C), respectively [32–33]. The signals observed at 287.2 eV (σ*: C–O and/or π^*^: C–OH), 288.4 eV (σ*: C–O), 290.1 eV (π^*^: C=O) and 291.6 eV (π^*^: O–C=O) correspond to a state in which the local sp^2^ bonding is influenced mainly by oxygen functionalization [32–33]. The position of the peak and the intensity of the spectra differ for NiFe-GO and CoNiFe-GO, indicating different electronic structures and interactions around Ni, Fe, Co, and GO.

Figure 3e presents the XRD pattern of the samples. The reflections of nickel hydroxide were detected at 2θ of around 10.8° and 22.5° (following the JCDS database (38–715)), for NiFe-GO, CoNiFe-GO, CoNiFe, and CoNiFe-GO, respectively, confirming a typical pattern of layered double hydroxides (LDHs) [34]. The analysis showed that the addition of GO into both NiFe and CoNiFe induced the formation of a nickel hydroxide LDH, which was observed in the XRD spectra as the appearance of more intense nickel LDH reflections. No LDH reflections were detected for NiFe, which can be related either to the absence of a LDH structure or a too faint XRD signal due to the very thin NiFe layer (200 nm).

**Figure 4:**
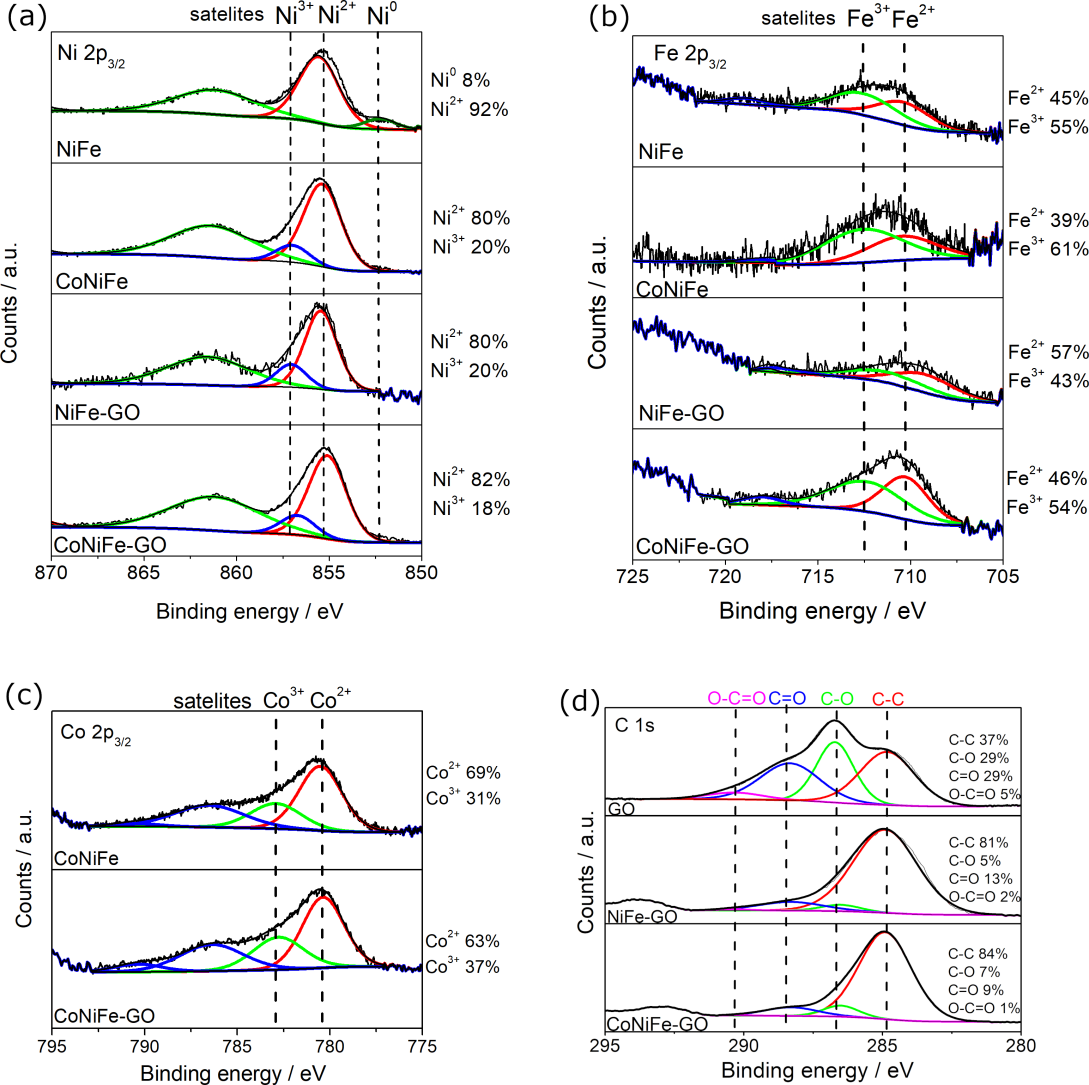
XPS high-resolution spectra of Ni 2p (a), Fe 2p (b), Co 2p (c), and C 1s (d) levels of the catalysts with the determined surface concentration of the elements (error ≤ 5%).

The XPS analysis showed that the addition of cobalt to NiFe induced the formation of the new nickel species Ni^3+^ in the catalyst structure (Figure 4a). The effect of the addition of cobalt to the NiFe on its structure was studied in detail in our previous work [25]. The appearance of Ni^3+^ was also observed after the addition of GO to NiFe. Both, GO and the addition of Co to NiFe resulted in the formation of nickel species in the oxidation states Ni^2+^ and Ni^3+^ with the same Ni^2+^/Ni^3+^ ratio of around 80%/20%. The addition of GO to CoNiFe did not change the structure of the catalyst concerning the type of the nickel species and the ratio of Ni^2+^/Ni^3+^ (80%/20%).

Two kinds of iron species were found in each of the materials studied, namely Fe^2+^ and Fe^3+^ (Figure 4b). The Fe^2+^/Fe^3+^ ratio in NiFe slightly decreased from 45%/55% to 39%/61% after the addition of cobalt. A different situation was observed in the case of the NiFe and CoNiFe catalysts after the addition of GO. The ratio of Fe^2+^/Fe^3+^ increased from Fe^2+^(45%)/Fe^3+^(55%) to Fe^2+^(57%)/Fe^3+^(43%) for NiFe, and from Fe^2+^(39%)/Fe^3+^(61%) to Fe^2+^(46%)/Fe^3+^(54%) for CoNiFe. The same type of cobalt species, that is, Co^2+^ and Co^3+^, and virtually the same percentage ratio of Co^2+^/Co^3+^ remained in the catalyst after the addition of GO to CoNiFe (Figure 4c).

Figure 4d presents the XPS spectra of the C 1s region of GO, NiFe-GO, and CoNiFe-GO. The C 1s spectrum of the catalysts indicates the degree of oxidation with four different components corresponding to carbon atoms in different functional groups, that is, non-oxygenated ring C–C (284.9 eV), the C in C–O (286.6 eV) and C=O (288.5 eV) bonds, and carboxylate carbon O–C=O (290.0 eV), which agrees with the XAS analysis (Figure 3) [35]. The analysis showed that the fraction of non-oxygenated ring C is about 37% for GO, while it increased significantly after combining GO with NiFe (81%) or CoNiFe (84%). The percentage of C–O, C=O, and O–C=O decreased down to around 5–7%, 9–13%, and 1–2%, respectively, for the GO-modified catalysts. The latter indicates that most of the oxygen functional groups in GO were removed, and thus the GO present in the structure of the NiFe or CoNiFe is in a reduced form [20]. The analysis confirms that the second step of the electrodeposition process leads to the simultaneous deposition of NiFe or CoNiFe and the reduction of the GO. A reduced form of GO combined with NiFe was also obtained by others after one-step electrodeposition by cyclic voltammetry [12].

### Electrochemical studies of the catalysts towards the OER

**Figure 5:**
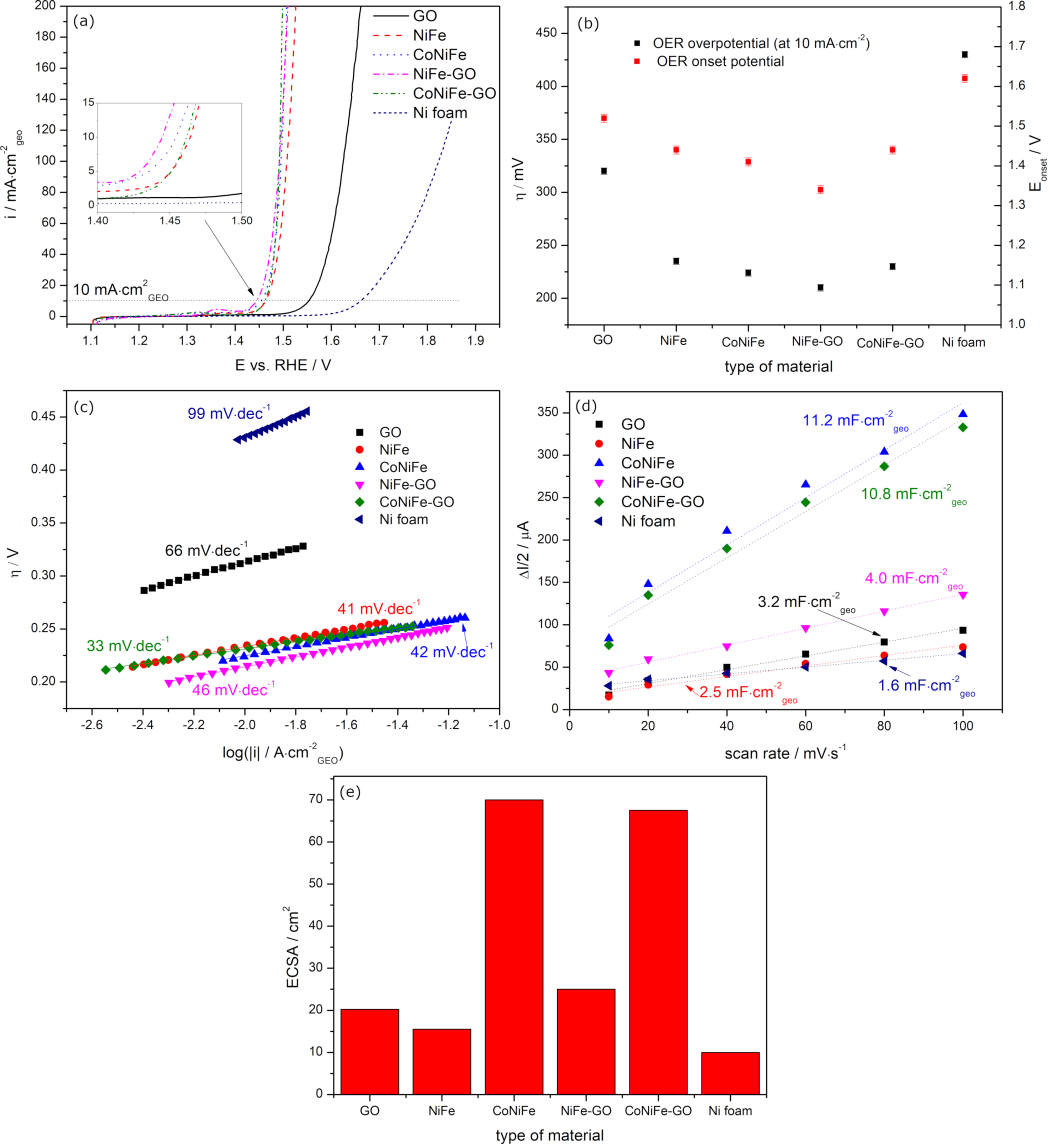
Linear scan voltammetry profiles (a) with corresponding evolutions of the OER overpotential η (10 mA·cm^−2^) and onset potential *E*_onset_ (b), and Tafel plots (c) of the catalysts. Double layer capacitance *C*_dl_ (d) and corresponding electrochemical active surface area (ECSA) (e) determined for each catalyst.

The electrochemical performance of the catalysts towards the OER was studied in an aqueous solution of 1 M KOH. Figure 5 presents the LSV graphs (Figure 5a) with the corresponding evolutions of OER overpotential (determined at 10 mA·cm^−2^), onset potential *E*_onset_ (Figure 5b), and Tafel plots (Figure 5c).

The LSV graphs and the corresponding evolutions of the OER overpotential and the onset potential *E*_onset_ show that coating the nickel foam with the catalyst layer resulted in each case in a higher catalytic performance of the sample towards the OER compared to the bare substrate. The addition of GO to NiFe significantly reduced η (10 mA·cm^−2^) and *E*_onset_ to 210 mV and 1.34 V, respectively, compared to GO (η: 320 mV, *E*_onset_: 1.52 V) and NiFe (η: 235 mV, *E*_onset_: 1.44 V) alone. A difference was observed in the case of the CoNiFe and CoNiFe-GO catalysts. Here, the addition of GO to CoNiFe (η: 230 mV, *E*_onset_: 1.44 V) significantly increased the OER catalytic activity of the sample compared to GO alone (η: 320 mV, *E*_onset_: 1.52 V), but the overall activity of the CoNiFe-GO was lowered compared to CoNiFe alone (η: 224 mV, *E*_onset_: 1.41 V).

The catalytic efficiency towards the OER can be also assessed by analyzing the Tafel plots of the catalysts (Figure 5c). The Tafel slope for bare nickel foam was determined to be 99 mV·dec^−1^, which is in agreement with the literature [36–37]. A lower Tafel slope was observed for nickel coated with GO, indicating faster kinetics towards the OER compared to the bare substrate [38]. The slopes for NiFe (41 mV·dec^−1^) and CoNiFe (42 mV·dec^−1^) were similar, which indicates that the same OER catalytic mechanism was in action. The addition of GO to NiFe resulted in a slight increase of the slope from 41 to 46 mV·dec^−1^, while the presence of GO in CoNiFe led to a decrease in Tafel slope down to 33 mV·dec^−1^.

Figure 5d and Figure 5e present a linear approximation of the capacitive currents as a function of the scan rate obtained from cyclic voltammograms with the determined double layer capacitance *C*_dl_ and the corresponding ECSA, respectively, for the samples. Coating the nickel foam with the catalysts resulted in each case in an increase of *C*_dl_/ECSA compared to the bare substrate. The highest value of *C*_dl_/ECSA was obtained for CoNiFe. The addition of cobalt to NiFe resulted in a nearly fourfold increase in the surface area of the catalyst. The latter was related to the change in morphology from the nanoplate-like structure typical for NiFe to the porous interconnected 3D nanoplate network typical for CoNiFe (Figure 1). The increase in the surface area of the catalyst after mild doping of NiFe with cobalt has also been described in the literature [39]. The addition of GO to CoNiFe left the value of *C*_dl_/ECSA of the material virtually unchanged (slightly lowered) compared to CoNiFe alone. A difference could be observed in the case of GO and NiFe. Here, the surface area of NiFe increased after adding GO to its structure. The value of *C*_dl_/ECSA of GO (3.2 mF·cm^−2^_geo_/20 cm^2^) is higher than that of NiFe (2.5 mF·cm^−2^_geo_/15.5 cm^2^) alone, which indicates that GO is responsible for the increase in the surface area of the NiFe-GO (4.0 mF·cm^−2^_geo_/25.0 cm^2^). The virtual lack of change in the CoNiFe-GO surface area and the change of the surface in the NiFe-GO sample compared to the catalysts alone may be due to the change in morphology observed in the SEM images (Figure 1).

**Figure 6:**
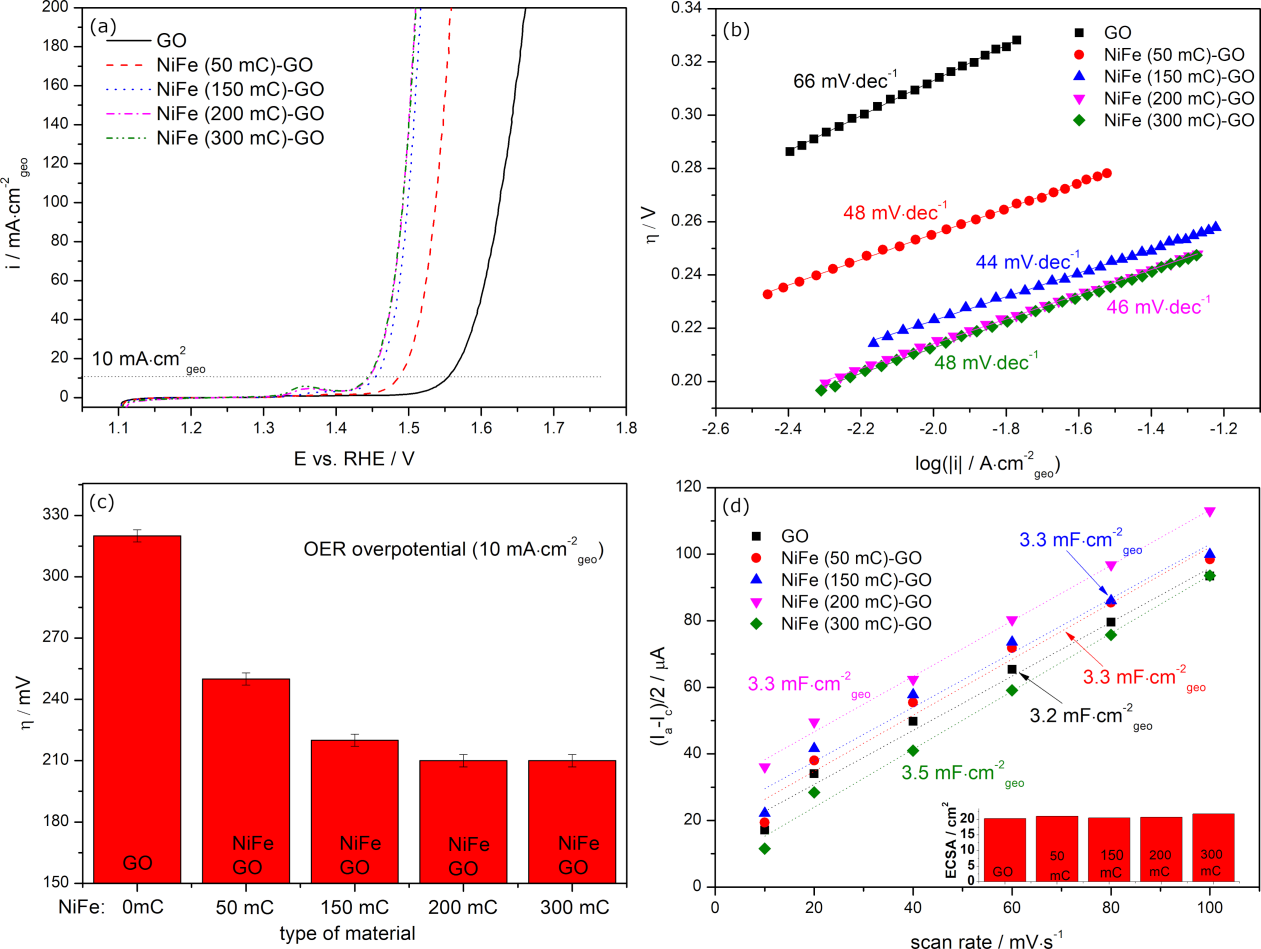
Linear scan voltammetry profiles (a) with corresponding Tafel plots (b) and evolution of the OER overpotential η(10 mA·cm^−2^) (c), and values of *C*_dl_ and ECSA (d) of the catalysts.

**Figure 7:**
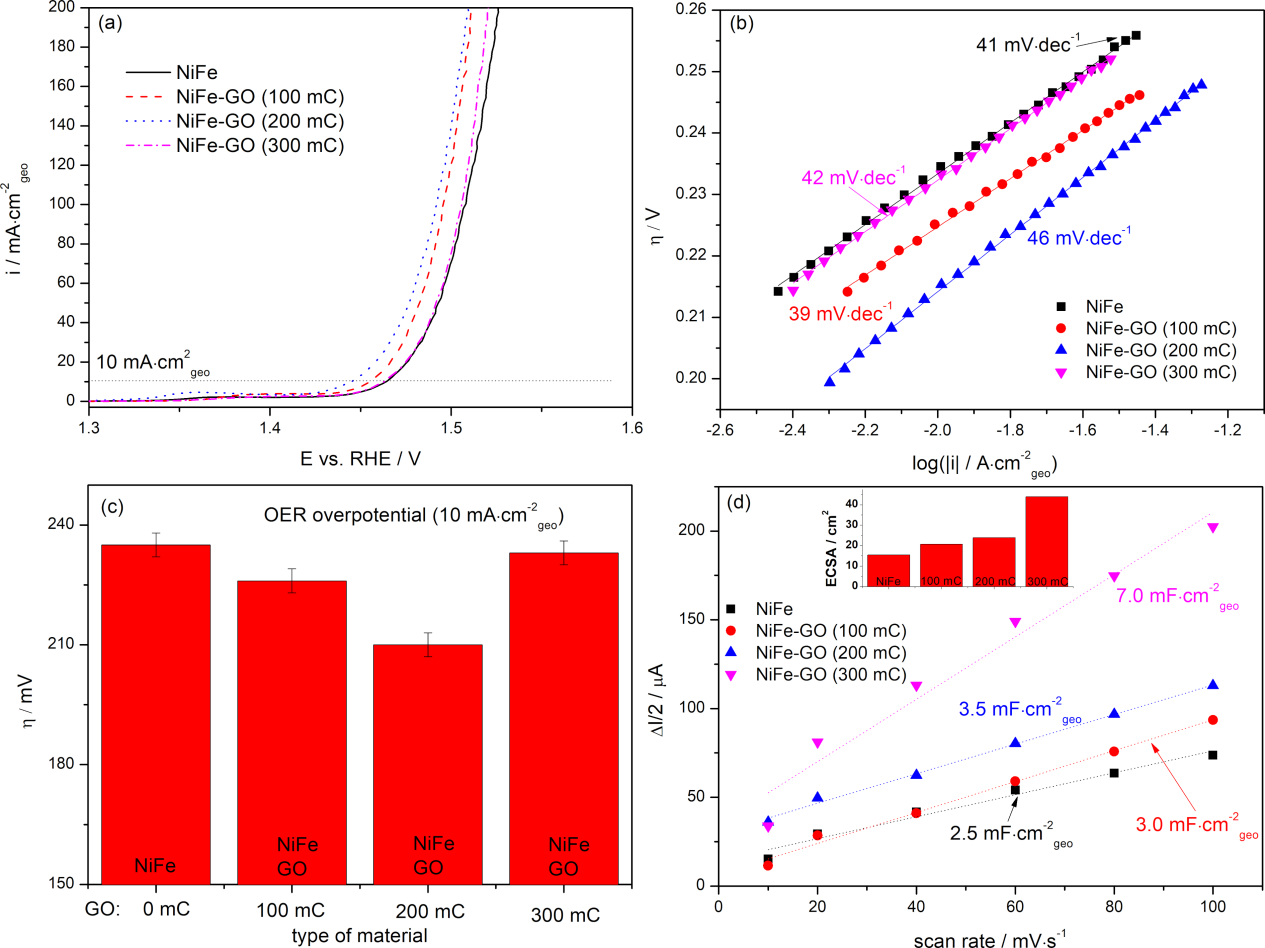
Linear scan voltammetry profiles (a) with corresponding Tafel plots (b) and evolution of the OER overpotential η (±3 mV) (c), and values of *C*_dl_ and ECSA (d) of the catalysts.

Since NiFe-GO revealed a higher catalytic activity towards the OER than NiFe and GO alone and the other catalysts, further electrochemical studies focused on this material. Figure 6 and Figure 7 present the effect of the change in the electrodeposition charge *Q*_dep_ of NiFe in NiFe-GO and GO in NiFe-GO, respectively, on their electrocatalytic performance towards the OER and on the value of *C*_dl_/ECSA. The LSV profiles of NiFe (*Q*_dep_: 50–200 mC)-GO recorded in an aqueous solution of 1 M KOH and the corresponding evolution of the OER overpotentials are presented in Figure 6a and Figure 6c, respectively. The graphs clearly show that the OER overpotential decreases with a higher deposition charge of NiFe, which is valid for *Q*_dep_ ≤ 200 mC. The lowest η(10 mA·cm^−2^), equaling 210 mV, was obtained for NiFe(200 mC)-GO, while the highest η(10 mA·cm^−2^) of 250 mV was obtained for NiFe(50 mC)-GO. The corresponding Tafel slopes reveal a similar trend as the OER η: the higher *Q*_dep_ of NiFe in NiFe-GO, the lower the slope (valid for *Q*_dep_ < 200 mC). Any change or deterioration of the OER catalytic activity of NiFe-GO for *Q*_dep_ > 200 mC may be due to the overgrow of deposited NiFe, which begins to block the ion and electron transport. The latter can also be confirmed by the Tafel slope analysis. The slopes for NiFe(300 mC)-GO begin to rise quickly, which indicates a change in the OER kinetics due to the slowed exchange of ions and electrons. The connection of GO with NiFe resulted in a slight increase of the value of *C*_dl_/ECSA compared to the GO and NiFe alone (Figure 5d,e). However, Figure 6d shows that this change is further independent on the NiFe deposition charge. A difference was observed for GO in NiFe-GO (Figure 7). The OER η of NiFe-GO(100–300 mC) decreased as the *Q*_dep_ of GO increased, which was valid for *Q*_dep_ ≤ 200 mC. A higher deposition charge of GO in NiFe-GO resulted in a re-increase of the OER η up to 233 mV, which was due to the overgrowth of GO over NiFe, characterized by a significantly higher value of *C*_dl_/ECSA of 7.0 mF·cm^−2^/44.0 cm^2^ compared to the rest of the samples.

This, in turn, resulted in blocking of the catalyst surface and the ion and electron transport became inhibited. The value of *C*_dl_/ECSA for NiFe-GO(100–300 mC) progressively increased as the deposition charge of GO in NiFe-GO increased, which was a different trend compared to NiFe(50–300 mC)-GO. Because of this, the data indicate that the improvement in the OER of NiFe-GO with the higher *Q*_dep_ of NiFe and GO resulted mainly from the NiFe structure and the electroactive surface area and the porosity of GO.

Electrochemical impedance spectroscopy (EIS) was performed in order to determine the charge transfer resistance (*R*_ct_) of the specific catalysts. The EIS spectra are presented in Supporting Information File 1 (Figure S4). *R*_ct_ values of 0.43, 0.50, 0.57, and 0.65 Ω were determined for NiFe-GO, CoNiFe, CoNiFe-GO, and NiFe, respectively. A decrease in *R*_ct_ is associated with more efficient reaction rates for the OER. The EIS results are in agreement with the trend of the evolution of η and *E*_onset_ determined based on LSV (Figure 5a,b).

**Figure 8:**
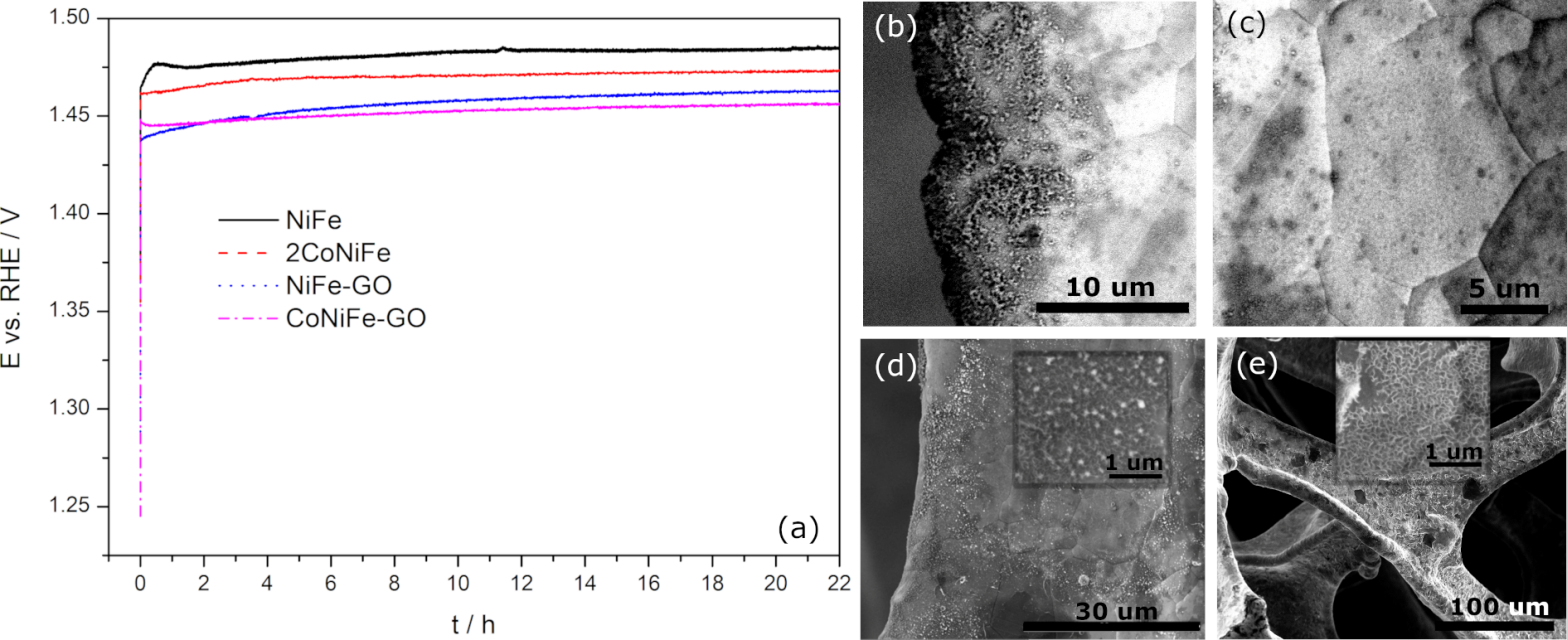
Chronopotentiometric curves recorded in aqueous solution of 1 M KOH at 10 mA∙cm^−2^_geo_ (a) and SEM images after the test of NiFe (b), CoNiFe (c), NiFe-GO (d), and CoNiFe-GO (e).

The stability test of the most promising among studied catalysts was assessed during chronoamperometry measurements at 10 mA∙cm^−2^_geo_ (Figure 8a).

A rapid increase of the potential at the beginning of the test was observed for each of the studied catalysts. This was due to the reduction of the catalysts’ active surface area through the physical absorption of the generated oxygen bubbles on the electrode surface, which was also observed by others [40]. The recorded potential deviated in a range of 1.46–1.48 V for NiFe, 1.46–1.47 V for CoNiFe, 1.44–1.46 V for NiFe-GO, and 1.44–1.45 V for CoNiFe-GO for the measurement period from *t*_0h_ to *t*_22h_. In each case, the presence of GO in the structure resulted in a lower working potential for the entire duration of the measurement compared to the catalysts without GO. Moreover, it can be also observed that the addition of cobalt in the structure of the catalysts resulted in a slightly lower potential deviation during the measurement from *t*_0h_ to *t*_22h_. The morphology of the catalysts after the test changed slightly in each case (Figure 8b–e). The plate-like structure remained for NiFe and CoNiFe catalysts. However, in each case, some material agglomeration occurred randomly on the surface of the electrodes. No detachment of the catalyst from the surface of the nickel foam was observed.

## Discussion

The studies showed that the addition of GO to NiFe and CoNiFe by electrodeposition significantly affected their morphological and structural properties, as well as on the electroactivity towards the OER. The addition of GO to NiFe resulted in a significant increase in the OER catalytic performance compared to GO and NiFe alone. OER η(10 mA·cm^−2^) and *E*_onset_ were reduced down to 210 mV and 1.34 V, respectively, compared to GO (η: 320 mV, *E*_onset_: 1.52 V) and NiFe (η: 235 mV, *E*_onset_: 1.44 V). A difference was observed for CoNiFe-GO. Here, the overall OER catalytic activity (η: 230 mV, *E*_onset_: 1.44 V) increased compared to GO alone. However, it was reduced in comparison with CoNiFe (η: 224 mV, *E*_onset_: 1.41 V). These phenomena can be associated with several factors. First, the morphology, which was changed when CoNiFe was combined with GO (Figure 1f). The morphology of CoNiFe-GO was characterized by a non-uniformly distributed 3D nanostructure of CoNiFe and some agglomerations of GO microflakes, which was not the case for GO and CoNiFe alone (Figure 1b and Figure 1d, respectively). A difference was observed for NiFe-GO, that is, the morphology of GO and NiFe remained virtually the same after the combination of the catalysts.

Another factor influencing the OER performance of the combined catalysts is the structure. The combination of GO with NiFe or CoNiFe resulted in the same oxidation states of nickel, iron, and cobalt in their structures (Figure 4). The addition of GO induced the formation of Ni^3+^ in NiFe-GO, so the final percentage ratio of Ni^2+^/Ni^3+^ in each of the studied catalysts became virtually the same (80%/20%) (Figure 4a). Also, the presence of GO induced the formation of Fe^2+^ in each of the catalysts. Thus, the ratio of Fe^2+^/Fe^3+^ in NiFe/CoNiFe-GO increased compared to the NiFe and CoNiFe. Moreover, it differed depending on the type of the catalyst, that is, it was 57%/43% for NiFe-GO and 46%/54% for CoNiFe-GO. The species in the oxidation state 3+ can be related to the presence of the (oxy)hydroxide form of the deposited catalysts, desirable for the OER process. The presence of a nickel (oxy)hydroxide LDH structure was confirmed by the XRD analysis. It was noticed that the addition of GO into the metallic structure induced the formation of a nickel hydroxide/(oxy)hydroxide LDH (Figure 3e). The electrochemical studies showed that the most efficient of the studied catalysts was NiFe-GO (η(10 mA·cm^−2^): 210 mV, *E*_onset_: 1.34 V). Thus, the presence of both nickel in the oxidation state 3+ and the LDH structure results in a more efficient OER reaction.

XAS analysis indicated the change in the electronic structure of the catalysts after the addition of GO (Figure 3). The analysis showed that the electronic structure around nickel and iron was changed, which may be associated with interactions between NiFe or CoNiFe and GO (carbon domains). Something similar was observed in the case of the addition of N-doped nanocarbon to NiFe [16]. To summarize, the disturbed morphology and the change in the electronic structure of CoNiFe after the addition of GO could result in a less attractive OER catalytic activity of this material compared to CoNiFe alone or NiFe-GO.

Further OER studies on NiFe-GO showed that, apart from the desirable morphology and structure, each of the materials forming the catalyst has a specific role in influencing the OER process. The increase of ECSA after combining metals with GO was only valid in the case of NiFe and GO. The addition of cobalt into NiFe caused a significant increase in ECSA, which resulted in a lower OER overpotential of CoNiFe, compared to NiFe. The latter was related to the change in the morphology from the nanoplate-like structure typical for NiFe to the porous interconnected 3D nanoplate network typical for CoNiFe (Figure 1). The addition of GO to CoNiFe left the value of *C*_dl_/ECSA of the material virtually unchanged (slightly lowered) compared to CoNiFe alone (Figure 5e). Because CoNiFe already revealed a very high ECSA, the addition of GO into CoNiFe lowered the catalytic OER activity compared to CoNiFe alone (Figure 5b). Most probably, the surface had become overloaded and some paths available for the reaction had been blocked. A different trend could be observed for NiFe and NiFe-GO. Here, the surface area of the NiFe increased after adding GO to its structure. The value of *C*_dl_/ECSA of GO (3.2 mF·cm^−2^_geo_/20 cm^2^) was higher than that of NiFe alone (2.5 mF·cm^−2^_geo_/15.5 cm^2^), which indicated that GO was responsible for the increase in the surface area of NiFe-GO (4.0 mF·cm^−2^_geo_/25.0 cm^2^). Therefore, the improvement in the OER with increasing *Q*_dep_ of the catalysts resulted mainly from the structure of NiFe (a change of *Q*_dep_ did not influence the ECSA, while the OER activity increased) and from the electroactive surface area of GO (a higher *Q*_dep_ resulted in a gradual increase of ECSA and OER activity).

## Conclusion

The effect of the addition of GO to electrodeposited NiFe and CoNiFe on their morphological and structural properties, as well as on the OER catalytic performance was studied successfully. The studies showed that modification of NiFe or CoNiFe with GO resulted in a significant change of structure, morphology, and OER activity. The changes differed depending on the presence of cobalt in the catalysts’ structure. The combination of GO with NiFe led to the formation of a uniformly deposited catalyst characterized by GO microflakes and NiFe nanoplates with higher values of *C*_dl_/ECSA (4.0 mF·cm^−2^/25.0 cm^2^) and OER activity (η: 210 mV, *E*_onset_: 1.34 V) compared to NiFe (*C*_dl_: 2.5 mF·cm^−2^, ECSA: 15.5 cm^2^, η: 235 mV, *E*_onset_: 1.44 V) and GO (*C*_dl_: 3.2 mF·cm^−2^, ECSA: 20.2 cm^2^, η: 320 mV, *E*_onset_: 1.52 V) alone. In contrast, the addition of GO to CoNiFe induced agglomerations of graphene flakes, which resulted in a slightly lower value of *C*_dl_/ECSA (10.8 mF·cm^−2^/67.5 cm^2^) and reduced OER activity (η: 230 mV, *E*_onset_: 1.44 V) compared to CoNiFe (*C*_dl_: 11.2 mF·cm^−2^, ECSA: 70 cm^2^, η: 224 mV, *E*_onset_: 1.41 V) alone. Further electrochemical studies on the most efficient catalyst NiFe-GO showed that a significant improvement in the OER catalytic activity was obtained from its specific structure, morphology, and electroactive surface area, obtained after the combination of NiFe and GO. It should be note that the main influences on the greater OER catalytic activity was the structure of NiFe and the electroactive surface area of GO.

## Experimental

### Fabrication of the catalysts

NiFe and CoNiFe oxides/(oxy)hydroxides were synthesized in a one-step process by electrodeposition at −1.1 V vs Ag/AgCl in an aqueous solution of 4 mM nickel(II) nitrate hexahydrate (Ni(NO_3_)_2_·6H_2_O) (98%, Sigma-Aldrich), 4 mM iron(III) nitrate nonahydrate (Fe(NO_3_)_3_·9H_2_O) (98%, Sigma-Aldrich), and 0 or 4 mM cobalt(II) nitrate hexahydrate (Co(NO_3_)_2_·6H_2_O) (98%, Sigma-Aldrich) at 25 °C. NiFe-GO and CoNiFe-GO were fabricated in a two-step process: (1) electrodeposition of GO performed at −1.0 V vs Ag/AgCl in an aqueous solution of 4.4 mg∙mL^−1^ GO (Graphene Supermarket) at 25 °C; (2) electrodeposition of NiFe or CoNiFe carried out at −1.1 V vs Ag/AgCl in an aqueous solution of 4 mM Ni(NO_3_)_2_·6H_2_O, 4 mM Fe(NO_3_)_3_·6H_2_O, and 0 or 2 mM Co(NO_3_)_2_·6H_2_O at 25 °C. Unless otherwise stated, the deposition time was limited to a charge of 200 mC for each deposition process. The deposition parameters, that is, the concentration of each metal nitrate and deposition charge, were optimized regarding the most efficient OER performance of the Ni-, Fe- and Co-based catalysts obtained in a previous work [25].

The electrodeposition was carried out in a one-compartment water-jacketed cell controlled by a potentiostat (VersaSTAT 4). The working electrode (WE) was nickel foam (MTI Corporation, purity > 99.9 wt %, surface density 346 g∙m^−2^, porosity ≥ 95%) or foil with an exposed area of 0.25 cm^2^. Before each deposition process, the substrates were cleaned ultrasonically in distilled water (5 min) and acetone (5 min). The reference and counter electrodes were Ag/AgCl (4 M KCl) and platinum mesh, respectively. Distilled water was used for the solutions. The measurement temperature was controlled by a thermostat (Julabo F12).

### Characterizations

The morphology and structure of the catalysts were characterized using a scanning electron microscope (FEI QUANTA FEG 250) with an energy-dispersive X-ray (EDX) sensor. X-ray absorption spectroscopy (XAS) was performed at the 04BM beamline at the National Synchrotron Radiation Centre SOLARIS [41]. The spectra were obtained using the total electron yield (TEY) detection mode, which can sample down to a depth of a few nanometers at room temperature. The beamline optics was optimized to perform the experiment with an energy resolution of 200 meV and better. X-ray diffraction (XRD) measurements were conducted using Cu Kα radiation (λ = 1.5404 Å) with a Philips X’Pert Pro diffractometer in the 2θ range from 5° to 35°. The selected 2θ range was selected based on the previous measurements corresponding to similar types of materials [25]. X-ray photoemission spectra (XPS) of the catalysts were obtained on an ultrahigh vacuum spectrophotometer at a pressure below 1.1 × 10^−8^ mbar at room temperature (Omicron NanoTechnology). Photoelectrons were detected by a spectrophotometer equipped with a 128-channel collector. The X-ray anode was operated at 15 keV and 300 W. The chemical composition calculations were determined based on the survey spectra collected in a wide range of binding energies, while valence state calculations were based on the high-resolution spectra. The C 1s peak (285.0 eV) was used to correct the results. Analysis of XPS spectra was performed with the Casa-XPS software using a Gaussian–Lorentzian (GL30) curve as a fitting algorithm and a Shirley background subtraction.

### Electrochemical studies

The setup for the electrochemical studies was the same as for the fabrication of the catalysts (see section “Electrosynthesis and morphology of the deposits”) with some exceptions: The working electrode was coated or bare nickel foam with an exposed area of 0.25 cm^2^, while the reference electrode was a reversible hydrogen electrode (RHE) (Gaskatel). The electrochemical cell was purged with argon for 20 min before each experiment. The measurements were performed in an aqueous solution of 1 M KOH (Stanlab, pH ≈13.9). Before each electrochemical experiment, the electrode was stabilized during cyclic voltammetry (CV) by sweeping the potential from 1.1 to 1.6 V vs RHE for at least 20 cycles with a scan rate of 100 mV·s^−1^. Linear scan voltammetry (LSV) was performed from 1.1 to 2 V vs RHE with a scan rate of 5 mV·s^−1^. The EIS spectra were recorded in the frequency range from 10 kHz to 1 Hz at 1.6 V vs RHE and amplitude of 10 mV. In order to determine *R*_ct_, EIS spectra were fitted with a simple Randles model with the solution resistance, charge transfer resistance, and the constant phase element (Zview). The OER stability test was carried out by chronoamperometry at a current density of 10 mA·cm^−2^ for 22 h. The recorded current values were normalized by the geometric area of the nickel electrode (0.25 cm^2^). All of the potentials were iR-corrected. The equation: η = *E*(10 mA·cm^−2^) − 1.23 V (vs RHE) was used to determine the OER overpotential [42]. The double-layer capacitance (*C*_dl_) was determined based on CV measurements, which were carried out within the potentials from 1.15 to 1.25 V vs RHE at a scan rate of 10, 20, 40, 60, 80, and 100 mV·s^−1^. Examples of the CV curves are presented in Figure S5, Supporting Information File 1. The following equation was used to determine *C*_dl_ from the CV: *C*_dl_ = *i*_dl_·(2ν)^−1^ = (*i*_a_ − *i*_c_)·(2ν)^−1^, where ν is the scan rate; *i*_a_ and *i*_c_ are the anodic and cathodic current densities, respectively, and *i*_dl_ is the double-layer current density. The electrochemical surface area (ECSA) was calculated based on the following equation ECSA = *C*_dl_·*A*·*C*_spec_^−1^, where *A* represents the geometric surface area of the sample and *C*_spec_ is the constant specific capacitance of 0.04 mF·cm^−2^_geo_, which is typical for a metallic electrode in an aqueous alkaline solution [43]. Each electrochemical experiment was performed a minimum of three times, and the average is presented in the manuscript.

## Supporting Information

File 1 Additional figures.
