# Supplementary material for: Evaluation of electrosynthesized reduced graphene oxide–Ni/Fe/Co-based (oxy)hydroxide catalysts towards the oxygen evolution reaction
Source: Beilstein J Nanotechnol. 2023 Mar 29;14:420–33. doi: 10.3762/bjnano.14.34 (PMC10071521; doi:10.3762/bjnano.14.34)
Supplement: File 1 — Additional figures. [file Beilstein_J_Nanotechnol-14-420-s001.pdf]

BEILSTEIN JOURNAL OF NANOTECHNOLOGY

Supporting Information

for

Evaluation of electrosynthesized reduced graphene oxide–Ni/Fe/Co-based (oxy)hydroxide catalysts towards the oxygen evolution reaction

Karolina Cysewska, Marcin Łapiński, Marcin Zając, Jakub Karczewski, Piotr Jasiński and Sebastian Molin

Beilstein J. Nanotechnol. **2023,** *14,* 420–433. doi:10.3762/bjnano.14.34

Additional figures

License and Terms: This is a supporting information file under the terms of the Creative Commons Attribution License (https://creativecommons.org/licenses/by/4.0). Please note that the reuse, redistribution and reproduction in particular requires that the author(s) and source are credited and that individual graphics may be subject to special legal provisions.
The license is subject to the *Beilstein Journal of Nanotechnology* terms and conditions: (https://www.beilstein-journals.org/bjnano/terms)

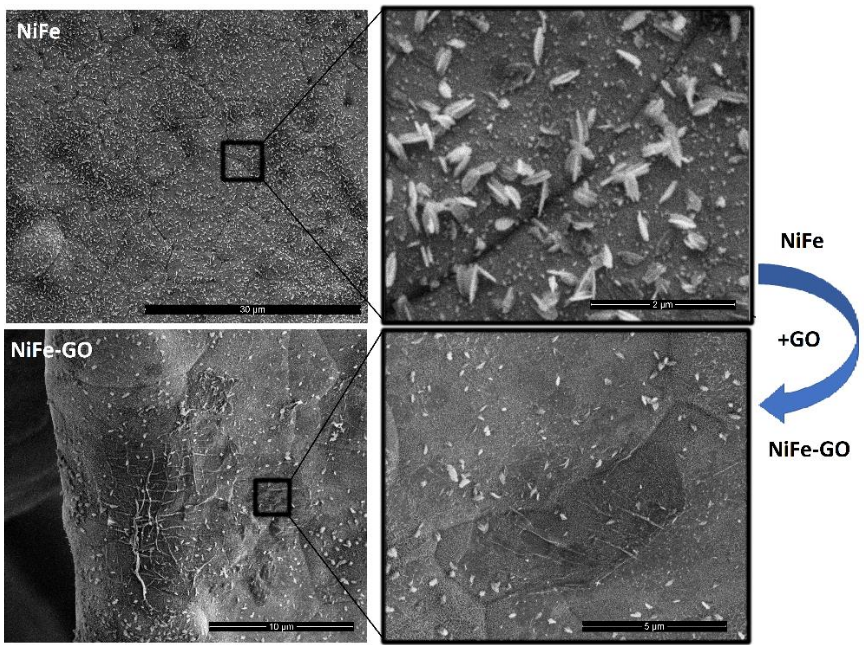

Figure S1: SEM images of NiFe and NiFe-GO.

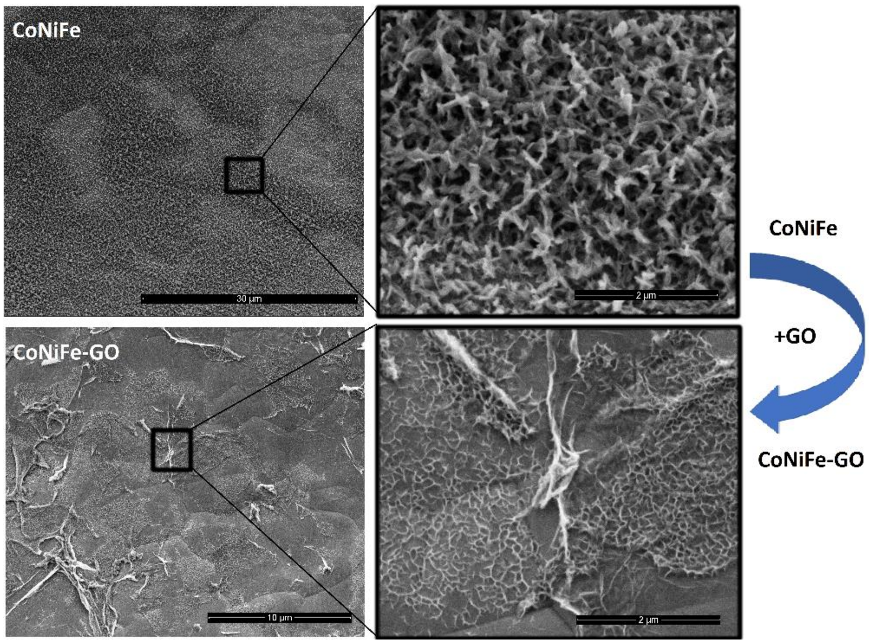

Figure S2: SEM images of CoNiFe and CoNiFe-GO.

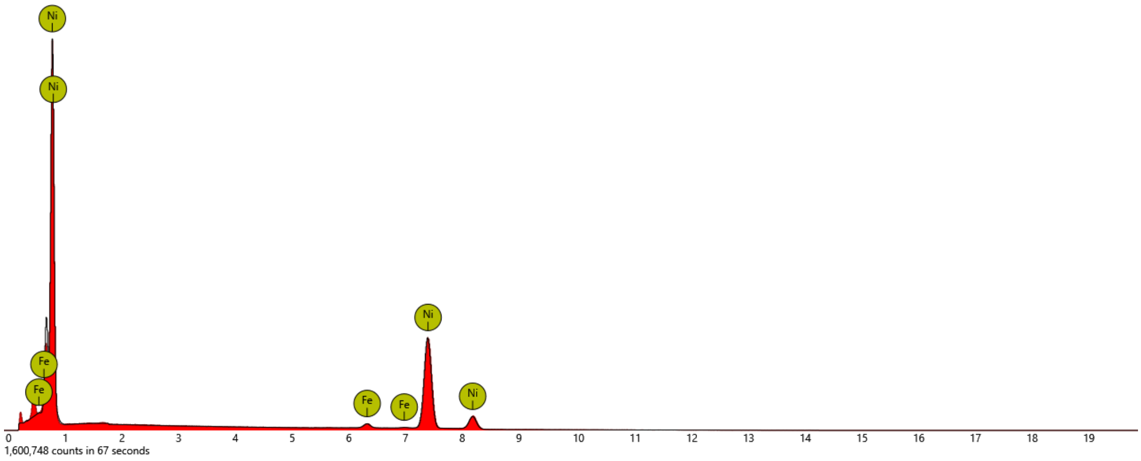

Figure S3: The content of the elements detected in NiFe-GO during EDX mapping.

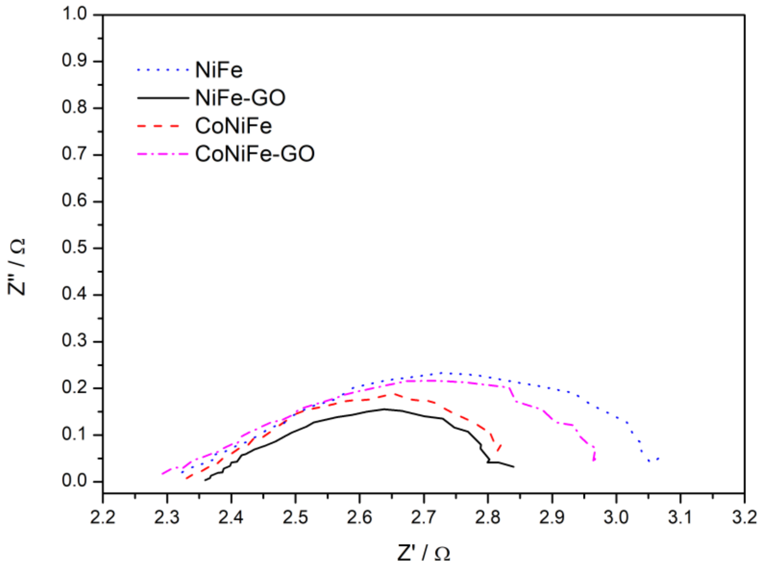

Figure S4: EIS spectra recorded at 1.6 V vs RHE in aqueous solution of 1 M KOH.

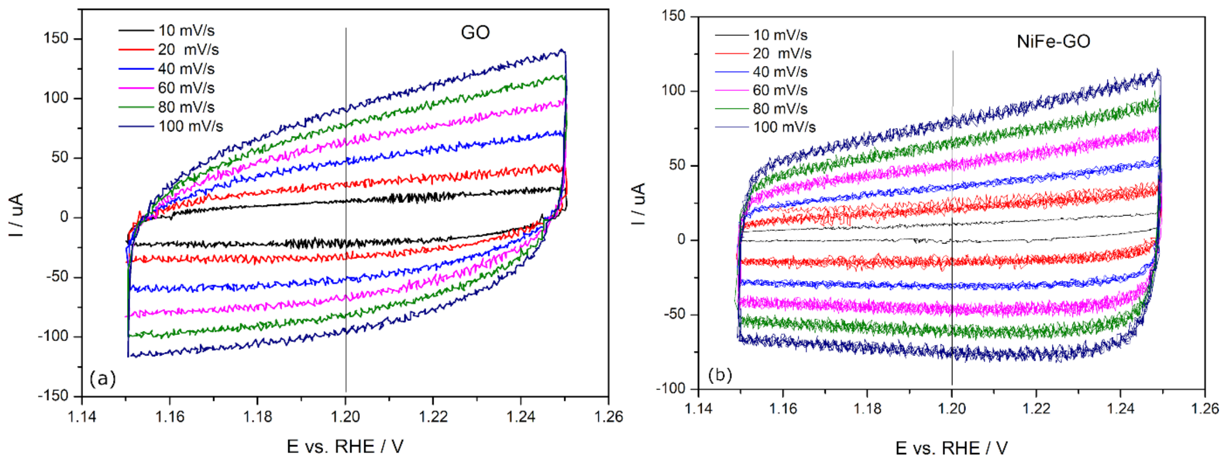

Figure S5: The example of cyclic voltammograms recorded during sweeping the potential from 1.15 to 1.25 V vs RHE with a scan rate of 10, 20, 40, 60, 80, and 100 $mV \cdot s^{-1}$ in aqueous solution of 1 M KOH for GO (a) and NiFe-GO (b). Similar graphs were obtained for the rest of the studied samples.
